# Improving gingival smile by means of guided bone regeneration principles

**DOI:** 10.1590/2177-6709.21.3.116-125.sar

**Published:** 2016

**Authors:** Carlos Eduardo de Almeida Ferreira, Roberto Carlos Bodart Brandão, Carolina Borges Martinelli, Túlio Bonna Pignaton

**Affiliations:** 1 Post-Doctoral Fellow, State University of New York, Buffalo, New York, USA.; 2 Professor of Orthodontics, Universidade Federal do Espírito Santo (UFES), Vitória, Espírito Santo, Brazil. Director of the Brazilian Board of Orthodontics and Facial Orthopedics (BBO).; 3 Masters student of Periodontology, São Leopoldo Mandic, Campinas, São Paulo, Brazil.; 4 Doctorate student of Implantodontics, Universidade Estadual Paulista (UNESP), Araraquara, São Paulo, Brazil.

**Keywords:** Bone regeneration, Maxillofacial development, Dental esthetics

## Abstract

**Objective:**

This study evaluated the effectiveness of guided bone regeneration (GBR) carried out with xenogenic bone substitute (Bio-Oss^TM)^ and collagen resorbable membrane (Bio-Gide^TM)^ to improve gingival smile (GS) in patients with excessive vertical maxillary growth (EVMG).

**Methods:**

Twelve healthy women aged between 20 and 49 years old (mean age of 26 years), with 5 mm or more of gingival exposure during fully posed smile (FPS) due to EVMG, were included. Baseline digital photographs were taken with standardized head position at rest and FPS. In eight out of 12 cases, crown lengthening procedure was indicated and the initial incision was made 2 to 4 mm from the gingival margin. In four cases, with no indication for crown lengthening procedure, a sulcular incision was performed. GBR was performed in all cases, using micro screws and/or titanium mesh associated with Bio-Oss^TM^ and Bio-Gide^TM^. After 10 days, sutures were removed. Recall appointments were scheduled at 1, 6, and 12 months when standardized photographs were again taken. ImageTool^TM^ software was used to measure the gingival exposure (GE) during FPS from the standardized close-up smile photographs at baseline and 12 months.

**Results:**

GE mean at baseline was 275.44 mm^2^. After 12 months, patients who undergone exclusively GBR procedure, presented GE reduction of 40.7%, ∆ = 112.01 mm^2^ (statistically significant, *p* = 0.12), and patients who had crown lengthening associated with the graft had a reduction of 60%, ∆ = 167.01 mm^2^.

**Conclusion:**

Our results using GBR to improve GS in cases of EVMG showed an exceptionally high patient acceptance and satisfaction. One-year follow-up confirmed stable results.

## RESUMO

**Objetivo:**

o presente estudo avaliou a eficácia da regeneração óssea guiada (ROG) usando enxerto ósseo xenógeno (Bio-Oss^(r))^ e membrana de colágeno reabsorvível (Bio-Gide^(r))^ para melhorar a estética do "sorriso gengival" (SG) em pacientes com crescimento maxilar vertical excessivo (CMVE).

**Métodos:**

foram incluídas 12 mulheres saudáveis com idades entre 20 e 49 anos (média = 26 anos) com 5mm ou mais de exposição gengival durante o sorriso amplo posado (SAP), em função de CMVE. Foram realizadas fotografias digitais padronizadas (FDP) em repouso e em SAP. Em 8 dos 12 casos, foi indicado o procedimento de aumento da coroa clínica, e a incisão inicial foi realizada de 2 a 4mm da margem gengival. Em 4 casos, para os quais não houve indicação de aumento da coroa clínica, foi realizada uma incisão intrassulcular. A ROG foi realizada, em todos os casos, utilizando-se mini-implantes e/ou malha de titânio, associados a Bio-Oss^(r)^ e Bio-Gide^(r)^. Após 10 dias, as suturas foram removidas. O programa ImageTool^(r)^ foi utilizado para medir a "área gengival exposta" (AGE) durante o SAP, nas imagens padronizadas do sorriso, em *close-up*, ao início do estudo e após 12 meses.

**Resultados:**

a média da AGE no início do estudo foi de 275,44mm^2^. Após 12 meses, os pacientes submetidos exclusivamente ao procedimento de ROG apresentaram redução na AGE de 40,7%, Δ = 112,01mm^2^ (estatisticamente significativo, p = 0,12); já nos pacientes em que o aumento de coroa clínica foi associado ao enxerto, a redução foi de 60%, Δ = 167,01mm^2^.

**Conclusão:**

os resultados do uso da ROG para melhorar o SG em casos de CMVE demonstraram aceitação e satisfação extremamente elevadas por parte dos pacientes. O acompanhamento de 1 ano pós-tratamento confirmou a estabilidade dos resultados.

## INTRODUCTION

The appearance of the smile is clearly of substantial importance and often one of the key criteria by which patients judge the success of their own treatment.^1^

With the increased emphasis on facial esthetics, both patients and dentists are developing a greater awareness of the impact of gingival display on the beauty of smile. The varied nomenclature for gingival smile (GS) includes "gummy smile," "high lip line," "short upper lip" and "full denture smile." In a group of 454 dental and dental hygiene students, Tjan et al found that 11% had high smile.^2^ The literature has shown that increased gingival display at smiling has worse esthetic evaluation by dentists and laypeople.^3^ ^,^ ^4^ ^,^ ^5^

To accurately diagnose and treat GS, the clinician must be able to recognize its different causes. The smile exhibiting gingival excess can be caused by altered passive eruption, dentoalveolar extrusion, vertical maxillary excess or a combination of these.^6^

Altered passive eruption, clinically presented by short clinical crowns, can be efficiently treated by periodontal crown lengthening surgical procedures.^7^ ^,^ ^8^

In some cases, orthodontic intrusion of maxillary anterior teeth with significant reduction of overjet and overbite may achieve slightly differences in the smile line.^9^ ^,^ ^10^

The most severe cases of gingival display are caused by excessive vertical development of the maxilla, also known as hyperdivergent face, idiopathic face, long face syndrome, vertical maxillary excess and long face.^9^ ^,^ ^10^

The most effective treatment for GS associated with maxillary vertical excess includes maxillary repositioning surgery (Le Fort I osteotomy) combined with orthodontic therapy. This method has its limitations and requires hospitalization and general anesthesia.^9^ ^,^ ^10^

As described in plastic surgery literature, soft tissue surgeries carried out to improve GS have been shown to be extremely unstable and unpredictable. Frequently, the reasons for disappointing results include treatment modalities incapable of addressing the basic problem: maxillary vertical excess.^11^

## OBJECTIVE

The aim of this study is to evaluate the effectiveness of guided bone regeneration (GBR) carried out by means of xenogenic bone substitute (Bio-Oss^TM)^ and resorbable membrane (Bio-Gide^TM)^ to improve GS in patients with excessive vertical maxillary growth (EVMG).

## MATERIAL AND METHODS

Twelve healthy women who refused undergoing orthodontic/orthognatic treatment, aged between 20 and 49 years old (mean 26 years), with 5 mm or more of gingival exposure during full posed smile (FPS) due to EVMG, were asked to perform full smile during preliminary examination until they were able to reproduce FPS three times. Baseline digital photographs were taken with standardized head position at rest and FPS. 

**Figure 1:**
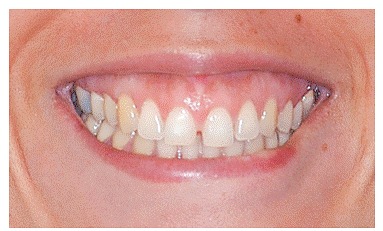
Initial smile.

Bone graft mock-up was done by placing a piece of cotton (Figs 1 to ^4^) under the patient's lip, in the vestibule, above the apex of teeth, extending from the nasal cavity to second premolars on both sides. The improvement was shown to patients.

**Figure 2:**
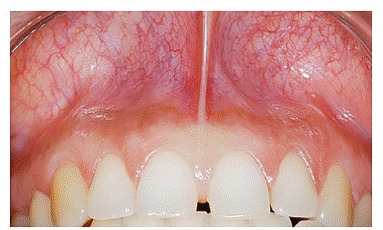
Vestibule under upper lip.

**Figure 3:**
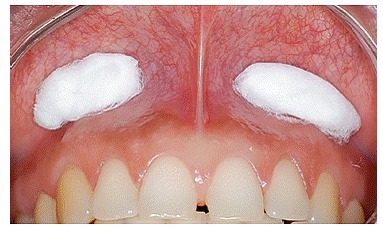
Piece of cotton used to simulate graft volume (mock-up).

**Figure 4:**
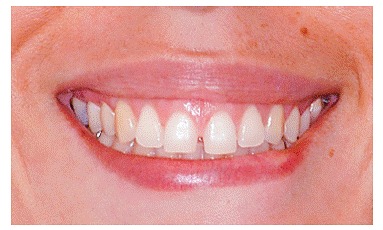
Smile after mock-up.

After mock-up approval, 12 subjects were screened for the study. Patients were asked to fill up a complete medical history form and presented no contraindication for surgical procedures. 

**Figures 5-14 - 5):**
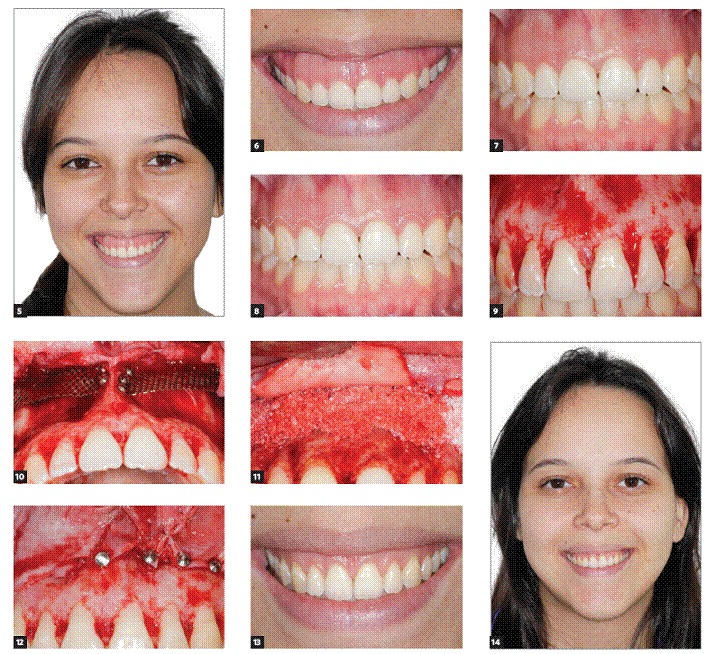
Initial frontal face view. 6) Initial smile view. 7) Initial intraoral view. 8) Illustration of initial incision in cases with indication of crown lengthening procedure performed 2 to 4 mm from the gingival margin. 9) Full thickness flap was raised, exposing the bony area between the apex of teeth and the nasal cavity along the lateral sinus wall. 10) Titanium mesh placed to keep the space under the membrane. 11) Space filled with anorganic bovine bone (Bio-Oss^TM)^. 12) A collagen membrane (Bio-Gide^TM)^ was used to cover the mesh and anorganic bovine bone (Bio-Oss^TM)^, stabilized with tacks. 13) Smile view 12 months after surgery. 14) Facial frontal view 12 months after surgery.

Initial frontal face, smile and intraoral photographs were taken and the surgery scheduled. Figures 5 to 7 show a select case.

After local anesthesia, in eight out of 12 cases with indication for crown lengthening, the initial incision was made 2 to 4 mm from the gingival margin. In four cases, with no indication for crown lengthening, a sulcular incision was performed. In both cases, procedures were carried out from the right maxillary molar to the equivalent molar on the left side (Fig 8). Vertical incisions were usually performed at the mesial surface of second molars. A full thickness flap was raised, exposing the bony area between the apex of teeth and the nasal cavity along the lateral sinus wall (Fig 9). By means of GBR principles, bone perforations were carried out and micro screws and/or titanium mesh were placed to keep the space under the membrane (Fig 10). The space was filled with Bio-Oss^TM^ (Geistlich, Germany) (Fig 11) and covered with Bio-Gide^TM^ (Geistlich, Germany), in addition to being stabilized with tacks (Fig 12). The flap was sutured to the original position. Patients were asked to take amoxicillin (500 mg, three times a day for seven days) and to rinse the site with 0.12% chlorhexidine for 10 days, at which point the sutures were removed.

**Figure 15:**
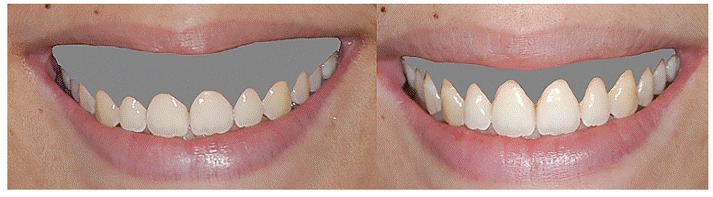
Illustration of gingival exposure (GE) measurement, before surgery and after one year. Note that the gingival band removed by means of the crown lengthening procedure was not considered as improvement for this measurement.

Recall appointments were scheduled at 1, 6 and 12 months when standardized extraoral photographs were again taken, as previously described. Figure 13 and ^14^ show facial frontal view and smile 12 months after surgery. A CT scan was also taken one year later. The University of Texas Health Science Center at San Antonio's free ImageTool^TM^ software was used to measure the gingival exposure (GE) during FPS from the standardized close-up smile photographs at baseline and 12 months after surgery. Figure 15 illustrates measurement before and one year after surgery. 

## RESULTS

**Figure 16:**
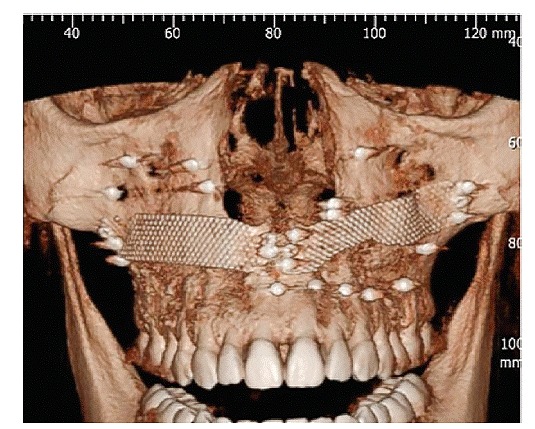
CT scan shows accommodation of titanium mesh and Bio-Oss^TM^ in the grafted area.

GE mean was 275.44 mm^2^ at baseline. Patients who underwent surgical procedure exclusively by GBR had a GE decrease of ∆ = 112.01mm^2^. This result represents an improvement of 40.7% in GS and was statistically significant (p = 0.12). The CT scan showed the accommodation of titanium mesh and Bio-Oss^TM^ in the grafted area (Fig 16). When crown lengthening was associated with graft, the mean improvement was ∆ = 167.01mm^2^ which represents a GS reduction of 60%.

Figures 17 and ^18^ show the improvement obtained exclusively by graft and the titanium mesh placement after one year. Figures 19 to 22 and 23 to 26 show two more cases with an even better result due to the combination of crown lengthening procedure with graft.

**Figures 17, 18:**
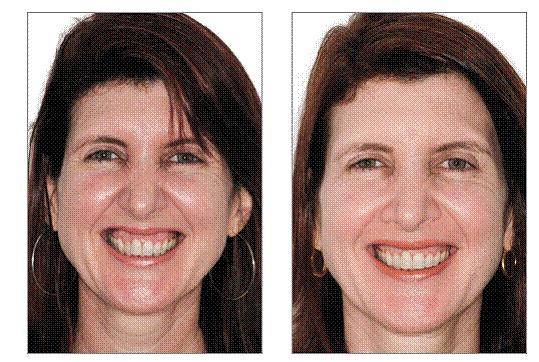
Improvement achieved exclusively by graft and titanium mesh placement after one year.

**Figures 19-26:**
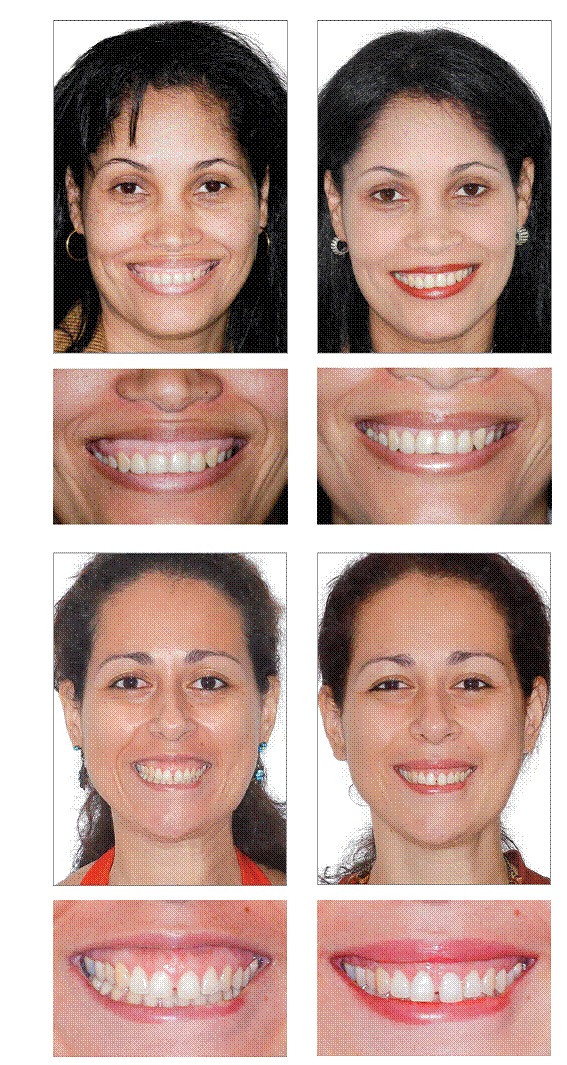
Two cases with an even better result due to the combination of crown lengthening procedure.

**Figures 27-36 27):**
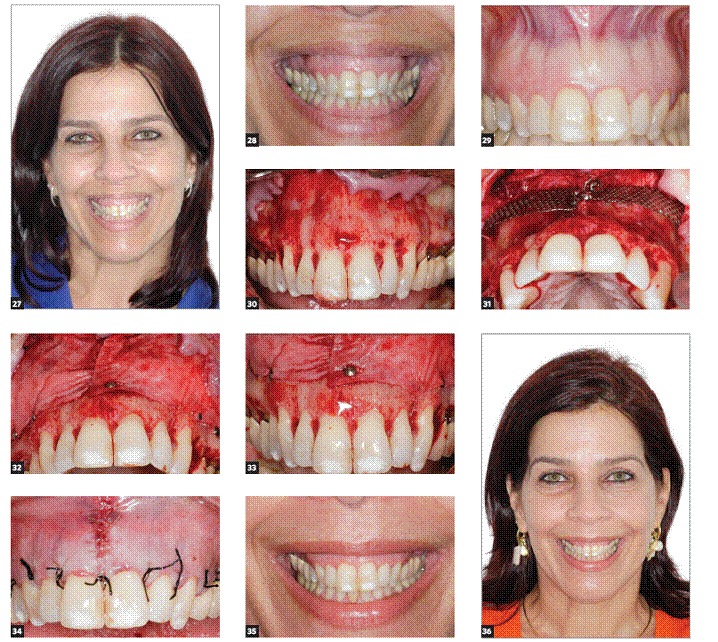
Initial frontal face view. 28) Initial smile view. 29) Initial intraoral view. 30) Full thickness flap was raised, exposing the bony area between the apex of teeth and the nasal cavity along the lateral sinus wall. 31) Titanium mesh placed to keep the space under the membrane. 32 and 33) Collagen membrane (Bio-Gide^TM)^ used to cover the mesh and anorganic bovine bone (Bio-Oss^TM)^, stabilized with tacks. 34) Flap replaced with tension-free sutures. 35) Smile view 12 months after surgery. 36) Facial frontal view 12 months after surgery.

Figures 27 to ^42^ show a complete clinical case sequence performed exclusively by graft and titanium mesh placement.

**Figure 37-42 37):**
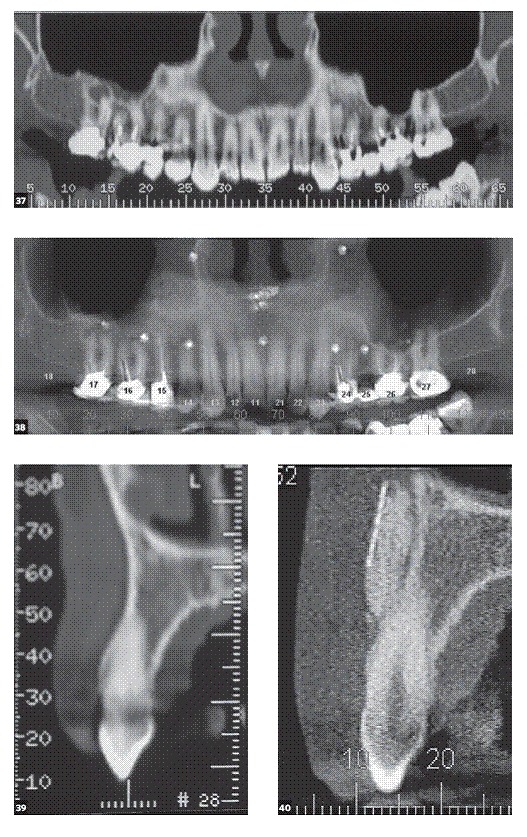
Panoramic view obtained from CT scan taken before surgery. 38) Panoramic view obtained from CT scan after surgery, showing titanium mesh and tacks. 39) Cross sectional image of #13 before surgery. 40) Cross sectional image of #13 after surgery. Note de space above de apex, grafted with Bio-Oss^TM^ under the titanium mesh. 41) 3D reconstruction obtained from CT scan before surgery. 42) 3D reconstruction obtained from CT scan shows accommodation of the titanium mesh and Bio-Oss^TM^ in the grafted area.

## DISCUSSION

The appearance of the smile is clearly of substantial importance and often one of the key criteria by which patients judge the success of their own treatment.^1^

To accurately diagnose and treat GS, the clinician must be able to recognize the different clinical presentations.^6^

Excessive gingival display was first published by Karin Willmar in 1974 ^12^ and is frequently caused by skeletal deformity that involves vertical maxillary excess, insufficient clinical crown length or a combination of both.^13^

Excessive vertical development of the maxilla generally produces exorbitant teeth display and gingival smile.^11^ The effective correction of this problem should be orthognatic surgery combined with orthodontic treatment. Hospitalization, general anesthesia and costs are the main limitations of this technique.

The first alternative procedure to treat GS was originally described by Rees and LaTrenta in 1989, when they addressed the hyperfunction of upper lip elevator as the main cause of the problem.^14^

The efficacy of camouflage lip lengthening procedures is doubtful. Soft tissue surgeries, such as the mucosa exclusion from the upper sulcus, frequently fail in long-term results, in addition to being incapable of addressing the basic problem: vertical maxillary excess.^11^

Botulinum toxin has also been recently described as a method for GS temporary improvement.^15^ ^,^ ^16^

Although in long face syndrome patients the upper lip seems to be short, cephalometric studies confirm that the upper lip is actually of normal length.^11^

Frequently, aging is followed by a decrease in gingival display at smile.^17^ Investigations of craniofacial dimensions demonstrate that significant changes occur in men, even during adult life.^18^ That is the reason why control photographs were taken one year after surgical procedures in our study. A longer follow-up to evaluate surgical improvement could be bias, considering the aging effect.

The most skeletal discrepancy on gingival smile line is probably the region above the apex of teeth, corresponding to the area from second premolar to lateral incisor. Only tridimensional tomography will be able to reveal skeletal discrepancies.^11^

In our study, the GBR procedure was able to fill up the bony cavity above the teeth apex, resulting in 40.7% improvement in gingival display. When the crown lengthening procedure was combined with graft, improvement was even better (60%).

Considerable variation exists in the literature regarding the postoperative time necessary to establish the final gingival levels after crown lengthening.^6^

Capturing patients's smiling images by means of digital photography has major drawbacks, e.g.: it is difficult to standardize the photographs due to differences in camera angles, distance from/to the patient, head position and discrepancies between intraoral and extraoral photographic techniques. 

This technique is not meant as a substitute for correction of severe vertical maxillary excess, but as a camouflage procedure with a remarkable improvement of 40.7% to 60% in gingival display in cases combined with a crown lengthening procedure.

## CONCLUSION

Results achieved by means of guided bone regeneration carried out to improve gingival smile in cases of long facial height showed high patient acceptance and satisfaction. One-year follow-up confirmed stable results. Controlled studies with a larger sample size should be planned for the near future.
